# Effect Sizes in Experimental Pain Produced by Gender, Genetic Variants and Sensitization Procedures

**DOI:** 10.1371/journal.pone.0017724

**Published:** 2011-03-10

**Authors:** Alexandra Doehring, Nele Küsener, Karin Flühr, Till J. Neddermeyer, Gaby Schneider, Jörn Lötsch

**Affiliations:** 1 pharmazentrum frankfurt/ZAFES, Institute of Clinical Pharmacology, Goethe-University, Frankfurt am Main, Germany; 2 Department of Computer Science and Mathematics, Goethe-University, Frankfurt am Main, Germany; Biological Research Center of the Hungarian Academy of Sciences, Hungary

## Abstract

**Background:**

Various effects on pain have been reported with respect to their statistical significance, but a standardized measure of effect size has been rarely added. Such a measure would ease comparison of the magnitude of the effects across studies, for example the effect of gender on heat pain with the effect of a genetic variant on pressure pain.

**Methodology/Principal Findings:**

Effect sizes on pain thresholds to stimuli consisting of heat, cold, blunt pressure, punctuate pressure and electrical current, administered to 125 subjects, were analyzed for 29 common variants in eight human genes reportedly modulating pain, gender and sensitization procedures using capsaicin or menthol. The genotype explained 0–5.9% of the total interindividual variance in pain thresholds to various stimuli and produced mainly small effects (Cohen's d 0–1.8). The largest effect had the *TRPA1* rs13255063T/rs11988795G haplotype explaining >5% of the variance in electrical pain thresholds and conferring lower pain sensitivity to homozygous carriers. Gender produced larger effect sizes than most variant alleles (1–14.8% explained variance, Cohen's d 0.2–0.8), with higher pain sensitivity in women than in men. Sensitization by capsaicin or menthol explained up to 63% of the total variance (4.7–62.8%) and produced largest effects according to Cohen's d (0.4–2.6), especially heat sensitization by capsaicin (Cohen's d = 2.6).

**Conclusions:**

Sensitization, gender and genetic variants produce effects on pain in the mentioned order of effect sizes. The present report may provide a basis for comparative discussions of factors influencing pain.

## Introduction

Pain is a complex and multi-factorial [1] trait and influenced by various and heterogeneous factors such as gender [2], genetic [3] or environmental causes [4]. Individual differences in pain responses [5] have been employed as a research tool of nociceptive or nocifensive mechanisms and are contemplated as a basis for personalized therapy approaches to pain. The multitude of factors modulating pain suggests a comparative assessment of their influences.

However, an experiment has a statistically significant effect, but also the size of any observed effects. In practical situations, effect sizes are helpful for making decisions. Although various effects on pain have been reported with respect to their statistical significance, a standardized measure of effect size has been rarely added. Such a measure would ease comparison of the magnitude of the effects across studies, for example the effect of gender on experimental heat pain with the effect of a genetic variant on pressure pain or clinical pain estimates. Reporting effect sizes is considered good practice when presenting empirical research findings in many fields [6]. In the present analysis, the effect sizes of factors currently of interest as modulators of pain, i.e., common genetic variants reportedly modulating pain (**Table 1**), gender [2], [7], [8] and sensitization procedures by capsaicin [9] or menthol [10] are provided.

**Table 1:** Effect sizes, expressed as percentage of the total variance explained by the genetic factors, on pain thresholds.

Factor	Polymorphism (dbSNP database number)	Ref.	MAF [%]	Effect sizes on pain thresholds (percentage explained variance of total variance), recessive hereditary model				
Genotype				Von Frey	Heat	Cold	Blunt pressure	Electric
*OPRM1* (μ-opioid receptor)	rs1799971 A>G	[28], [50]	9.2	0.35	0.76	0.08	1.74	1.43
*OPRD1* (δ-opioid receptor)	rs1042114 T>G	[29]	17.2	0.04	0.2	0.45	2.02	4.74
	rs2234918 T>C	[29]	44.4	0.25	0.79	0.04	1.14	0
*COMT* (Cathechol-O-methyl transferase)	rs4646312 T>C	[30]	36.8	1.08	0.77	0.47	0.44	0.04
	rs6269 A>G	[30]	37.6	0.77	*1.45*	0.01	0	0.21
	rs4633 C>T	[51]	54	2.33	0.2	0.32	1.16	1.17
	rs4680 G>A	[52], [53]	53.2	0.99	0.41	0.29	0.62	1.17
	rs6269G/rs4633C/4818G/rs4680G	[51]	36.4	0.77	*1.45*	0.01	0	0.21
	rs6269A/rs4633T/4818C/rs4680A		50.8	0.6	*1.05*	1.2	0.71	1.9
	rs6269A/rs4633C/4818C/rs4680G		8.4	-	-	-	-	-
	rs4646312T/rs165722T/rs6269A/rs4633T/rs4818C/rs4680A	[30]	49.6	1.08	*1.28*	0.55	0.18	1.41
	rs4646312C/rs165722C/rs6269G/rs4633C/rs4818G/rs4680G		34	0.51	0.5	0.03	0.08	0.23
	rs4646312T/rs165722C/rs6269A/rs4633C/rs4818C/rs4680G		7.6	-	-	-	-	-
*TRPV1* (Transient receptor potential cation channel, subfamily V, member 1)	rs8065080 A>G	[29]	36.8	0.27	0.18	0.01	0.04	0.11
*TRPA1* (Transient receptor potential cation channel, subfamily A, member 1)	rs11988795 G>A	[30]	32.8	1.02	0.06	0.26	0.01	0.26
	rs13255063A/rs11988795G	[30]	38.8	0.1	0.06	*1.42*	0.26	0.01
	rs13255063A/rs11988795A		32.8	1.02	0.06	0.26	0.01	0.26
	rs13255063T/rs11988795G		28.4	3.49	0.92	*3.74*	0.25	**5.91**
*FAAH* (Fatty acid amide hydrolase)	rs932816 G>A	[30]	23.6	0.03	0.06	0.06	1.26	0.56
	rs4141964 T>C	[30]	42.8	0.17	0.12	0.05	0.5	0.11
	rs2295633 G>A	[30]	41.6	0.47	0.32	0.02	0.09	0.01
	rs932816G/rs4141964T		34.4	1.37	*1.31*	0.81	0.08	0.26
	rs932816G/rs4141964C		42	0.04	0.31	0.03	0.01	0.11
	rs932816A/rs4141964T		22.8	0.03	0.06	0.06	1.26	0.56
	rs324419C/rs2295633G		58.4	0	0.13	0.01	1.2	0.58
	rs324419C/rs2295633A		22.4	0.01	0.12	0.27	0.2	0.33
	rs324419T/rs2295633A		19.2	1.3	0.14	0.99	0.72	0.99
*GCH1* (GTP cyclohydrolase 1)	1 particular haplotype of 3 SNPs associated to one of 15 SNPs	[41], [42]	16.4	1.08	*1.6*	0.89	0.45	0.63
*MC1R* (Melanocortin-1 receptor)	2 variant alleles of 29insA, 451C>T, 478C>T, 479G>A, 880G>C (“red head fair skin” phenotype, n = 2)	[54]	451T: 6.4%, 478T: 6%, others: 0–0.4%	0.01	*1.09*	0.47	0	0

# MAF: Observed minor allelic frequencies. “Minor” refers to the allele reported to be minor in gene databases. When its reported allelic frequency is close to 50%, it can happen that the “minor” allele has a frequency >50% in the actual cohort. We nevertheless preserved the denomination “minor” to be consistent with SNP databases.

The reference and the observed allelic frequencies are given, and the recessive hereditary model was used, i.e., assigning heterozygous subjects to the group of homozygous mutated carriers. The effect sizes are given in italic letters when they were larger than those of gender, and in bold letters when exceeding, arbitrarily chosen, 5%.

## Methods

### Subjects and design

The study was conducted following the Declaration of Helsinki on Biomedical Research Involving Human Subjects. The University of Frankfurt Medical Faculty Ethics Review Board approved the study protocol. Informed written consent was obtained from all subjects. Pain thresholds to various experimental stimuli had been determined during previous assessments [11], [12] in a random sample of 125 unrelated healthy caucasian volunteers (69 men, 56 women, aged 18 to 46 years, mean 25±4.4 years). Exclusion criteria were drug intake dated back less than a week except for oral anticonceptionals, an actual clinical condition involving pain, and actual diseases according to questioning and medical examination. A training session was performed prior to the actual experiments, however, without application of sensitization procedures. Employing an open non-randomized design the actual measurements (for pain models, see the following section) took place in the order cold pain, menthol application, von Frey hair pain, cold/menthol pain, heat pain, capsaicin application, electrical pain, heat/capsaicin pain, pressure pain and von Frey hair/capsaicin pain, at intervals of 3–5 min between models.

### Assessment of pain

The study assessed pain thresholds to various stimuli defined as “the least experience of pain which a subject can recognize” (http://www.iasp-pain.org). Pain models were applied without knowledge of the genotypes. Five different stimuli were applied to include a broad variety of thermal, mechanical and electrical pain [11], [12]. In brief, **heat** stimuli were applied using a 3×3 cm thermode (Thermal Sensory Analyzer, Medoc Advanced Medical Systems Ltd., Ramat Yishai, Israel) placed onto the skin of the left volar forearm. Its temperature was increased from 32°C by 0.3°C/s until the subject pressed a button at the first sensation of pain, which triggered cooling of the thermode by approximately 1.2°C/s. Heat stimuli were applied eight times at random intervals of 25–35 s. The median of the last five responses was defined as the heat pain threshold because in previous experiments a plateau was reached after the first three measurements. **Cold** stimuli were applied at inner side of the right forearm, similarly to heat pain thresholds. The temperature was decreased from 32°C to 0°C by 1°C/s. As previous experiments had shown that measurements are stable from the first application, five repetitions were used and the threshold was the median of these measurements. **Blunt pressure** was exerted perpendicularly onto the dorsal side of mid-phalanx of the right middle finger using a pressure algometer with a circular and flat probe of 1 cm diameter (JTECH Medical, Midvale, USA). The pressure was increased at a rate of approximately 9 N/cm^2^ per second until the subject reported pain. The procedure was repeated five times at intervals of 30 s. Mechanical pain threshold to blunt pressure was the median of the five measurements. **Punctate pressure** was exerted onto the left volar forearm using von Frey hairs (0.008, 0.02, 0.04, 0.07, 0.16, 0.4, 0.6, 1, 1.4, 2, 4, 6, 8, 10, 15, 26, 60, 100, 180, 300 g; North Coast Medical Inc., Morgan Hill, CA, USA). Von Frey hairs were applied at randomized order and the pain threshold was the (log-transformed) turning point at 50% probability of a logistic regression of the “pain/no-pain” answers. During the experiments, subjects had to keep their eyes closed to avoid recognition of the von Frey hairs' strength. **Electrical** stimuli were applied using a constant current device (Neurometer® CPT, Neurotron Inc., Baltimore, MD). It delivered sine-wave stimuli at 5 Hz applied via two gold electrodes placed on the medial and lateral side of the mid-phalanx of the right middle finger. Their intensity was increased from 0 to 20 mA by 0.2 mA/s until the subjects interrupted the current by releasing a button. Measurements were repeated five times at intervals of 30 s and the median of these measurements was submitted to statistics as the electrical pain threshold. **Sensitization** was assessed with punctate mechanical and heat [9] stimuli and obtained using capsaicin cream (0.1 g, 0.1%, manufactured by the local pharmacy) applied onto a 3×3 cm skin area on the left volar forearm and covered with a plaster for 20 min. Sensitization to cold stimuli [10] was assessed using menthol solution (2 ml of a 40% menthol solution dissolved in ethanol) applied in a soaked plaster onto a 3×3 cm skin area on the right volar forearm for 20 min.

### Data analysis

To obtain the genotypes for this assessment, those single nucleotide polymorphisms (SNP) or haplotypes reported until June 2008 to modulate experimental pain in healthy average people (n = 29, Table 1) were diagnosed by means of validated Pyrosequencing^TM^ assays. Genotypes were submitted to further analysis after verifying that the distribution of homozygous and heterozygous carriers of variants was as expected from the Hardy-Weinberg [13] law (χ^2^ goodness-of-fit tests: p>0.05).

The physical strengths of the stimuli at which the subjects' answer to the question “Does it hurt?” changed from “No” to “Yes” were the pain threshold to the respective stimuli and were analyzed for the effect sizes of the genetic and non-genetic factors. The **portion of the total variance** in a pain threshold **explained** by a particular factor was calculated for every factor *j* (genetic variants including SNPs and *in-silico* obtained haplotypes [14], gender or sensitization by capsaicin or menthol application) as 
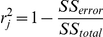
, where *SS* denotes the sum of squared deviations from the mean of the respective pain scores *j*, and the error SS describes the SS being not due to the genetic or gender factor. As sensitization involved repeated measurements, the variance explained was assessed using a resampling procedure without replacement that provided 1000 new data sets containing either the non-sensitized or sensitized thresholds from a single person, which allowed using sensitization as an inter-individual factor as gender or genetics.

In meta-analysis often performed to draw standardized information about effect sizes from heterogeneous data sets, the effect sizes are being quantified by calculating **Cohen's d** [15] as a widely used standardized effect size measure appropriate to use in the context of a t-test on means. Specifically, genotype effects can be assessed by means of t-tests, i.e., in the dominant hereditary model by comparing carriers with non-carriers of a variant, and in the recessive hereditary model by comparing homozygous carriers of a variant with pooled heterozygous and non-carriers. Standardized group differences in parameter means were calculated as 
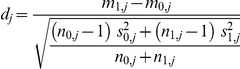
, where *m_0,j_*, *m_1,j_* and *s_0,j_*, *s_1,j_* denote the means and standard deviations of the pain scores in the carriers or non-carriers of the compared property *j*. The result is a unit-free number of which, an absolute value of d = 0.2 is regarded as a small effect, 0.5 as a medium and >0.8 as a large effect [15].

## Results

**Figure 1:**
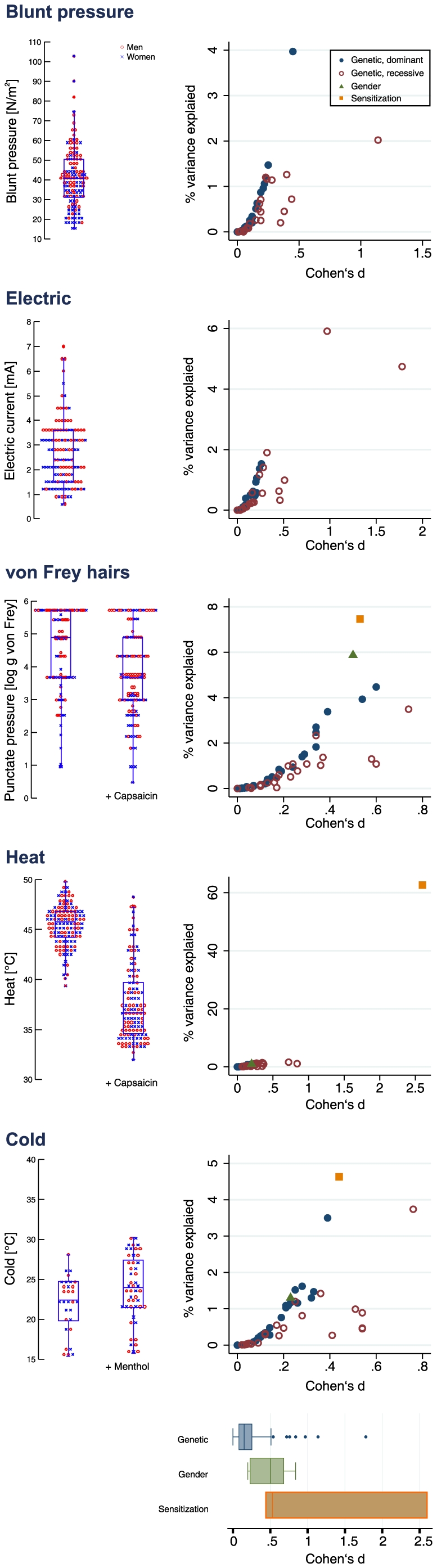
Observed thresholds to different pain stimuli and sizes of modulatory effects. **Left** part: Single values of the measured pain thresholds to various stimuli are shown as dots, with statistical summaries in overlaid box plots. The boxes span the 25^th^ to 75^th^ percentiles, with the median crossing the box as a horizontal line, and the whiskers spanning values within 1.5 times the 25^th^ to 75^th^ percentiles. The subject's gender is indicated by different symbols and colors (men: red circles, women: blue crosses). At the **right** of each thresholds presentation, the effect sizes of the genetic variants obtained using the dominant hereditary model (blue filled circles), i.e., heterozygous and homozygous carriers of the variant alleles versus wild type subjects, and the recessive model (red empty circles), i.e., homozygous carriers of the variant versus the other subjects, are shown as correlation plots between the fraction of the total variance in the respective threshold explained by the respective factor and Cohen's d of that factor. An absolute value of d = 0.2 indicates a small effect, values around 0.5 a medium and above 0.8 a large effect [15]. In addition, the effects sizes of gender (green filled triangles) and sensitization (orange filled squares) by capsaicin (heat, von Frey hair punctate pressure) or menthol (cold) are shown. Note that the axis scaling is non-uniform among panels to enhance data visibility. At the bottom, the overall effect sizes (all Cohen's d per condition genetics, gender or sensitization) of all analyzed factors and stimuli are grouped for genetic, gender and sensitization influences on pain thresholds, showing decreasing sizes of effects in the order sensitization, gender and genetics.

The original physical stimulus strengths at which the stimuli became painful are shown in Figure 1 (left panels). The different **genotypes** explained 0–5.9% (**Table 1** and >Table 2) of the variance in these pain thresholds to the different stimuli. For example, the *GCH1* haplotype explained 4% of the interindividual variance in pressure pain thresholds while the δ-opioid receptor variant rs1042114 explained 2.5% of the variance across subjects in von Frey hair thresholds. According to Cohen's d (Table 3 and Table 4), the genotype effect sizes had to be considered as mostly small (range 0–1.78). A gene dose effect resulted in somewhat larger effect sizes in homozygous carriers. However, only the *TRPA1* rs13255063T/rs11988795G haplotype explained >5% of the variance, namely of that in electrical pain threshold. Homozygous carriers had a higher pain threshold to electrical stimuli (3.8±2 mA versus 2.5±1.2 mA, p = 0.006).

**Table 2:** Effect sizes, expressed as percentage of the total variance explained by the respective factor, on pain thresholds.

Factor	Polymorphism (dbSNP database number)	Effect sizes on pain thresholds (percentage explained variance of total variance), dominant hereditary model				
Genotype		Von Frey	Heat	Cold	Blunt pressure	Electric
*OPRM1* (μ-opioid receptor)	rs1799971 A>G	0.21	0.56	0.28	0.2	0.14
*OPRD1* (δ-opioid receptor)	rs1042114 T>G	2.47	0.01	1.17	0.12	0.58
	rs2234918 T>C	0.76	0.72	0.76	0.13	0.06
*COMT* (Cathechol-O-methyl transferase)	rs4646312 T>C	0.4	0.06	0.34	0.1	0.18
	rs6269 A>G	0	0.86	*3.5*	1.47	1.53
	rs4633 C>T	0.94	*1.23*	0.09	0.15	0.01
	rs4680 G>A	1.83	0.46	0.02	0.03	0.12
	rs6269G/rs4633C/4818G/rs4680G	0.13	0.14	1.52	0.87	0.52
	rs6269A/rs4633T/4818C/rs4680A	1.4	0.71	0.12	0.02	0.01
	rs6269A/rs4633C/4818C/rs4680G	3.93	0	*1.47*	0	0.48
	rs4646312T/rs165722T/rs6269A/rs4633T/rs4818C/rs4680A	1.51	0.65	0.01	0.02	0.03
	rs4646312C/rs165722C/rs6269G/rs4633C/rs4818G/rs4680G	0.04	0	1.09	0.51	0.5
	rs4646312T/rs165722C/rs6269A/rs4633C/rs4818C/rs4680G	4.47	0.12	1.3	0.03	0.57
*TRPV1* (Transient receptor potential cation channel, subfamily V, member 1)	rs8065080 A>G	0.03	0.85	0.29	0.63	1.37
*TRPA1* (Transient receptor potential cation channel, subfamily A, member 1)	rs11988795 G>A	0.01	0.12	0.25	0.25	1.06
	rs13255063A/rs11988795G	0.1	0.25	0.22	0.31	0.93
	rs13255063A/rs11988795A	0.01	0.12	0.25	0.25	1.06
	rs13255063T/rs11988795G	0.84	0.12	0.2	0.01	0.1
*FAAH* (Fatty acid amide hydrolase)	rs932816 G>A	2.7	0.27	1.09	0.09	0.15
	rs4141964 T>C	0.02	0.27	0	1.06	0.62
	rs2295633 G>A	0	0.13	0.01	1.2	0.58
	rs932816G/rs4141964T	0.51	0.7	0.48	0.28	0.01
	rs932816G/rs4141964C	0.02	0.27	0	1.06	0.62
	rs932816A/rs4141964T	3.38	0.47	1.03	0.05	0.15
	rs324419C/rs2295633G	0.47	0.32	0.02	0.09	0.01
	rs324419C/rs2295633A	0.08	0.04	*1.62*	0.34	0.39
	rs324419T/rs2295633A	0.02	0.74	1.16	0.96	0.58
*GCH1* (GTP cyclohydrolase 1)	1 particular haplotype of 3 SNPs associated to one of 15 SNPs	0.38	0.3	0.35	3.97	0
*MC1R* (Melanocortin-1 receptor)	2 variant alleles of 29insA, 451C>T, 478C>T, 479G>A, 880G>C (“red head fair skin” phenotype, n = 2)					
**Gender**		**5.87**	0.95	1.3	**14.75**	**10.27**
**Sensitization**		***7.46***	***62.6***	*4.63*		

# MAF: Observed minor allelic frequencies. “Minor” refers to the allele reported to be minor in gene databases. When its reported allelic frequency is close to 50%, it can happen that the “minor” allele has a frequency >50% in the actual cohort. We nevertheless preserved the denomination “minor” to be consistent with SNP databases.

In the case of the genetic factors, the reference and the observed allelic frequencies are given, and the dominant hereditary model was used, i.e., assigning heterozygous subjects to the group of wild-type carriers. The effect sizes are given in italic letters when they were larger than those of gender, and in bold letters when exceeding, arbitrarily chosen, 5%.

**Table 3:** Effect sizes, expressed as absolute values of Cohen's d [15], of the genetics factors on pain thresholds.

Factor	Polymorphism (dbSNP database number)	Effect sizes on pain thresholds (Cohen's d), recessive hereditary model				
Genotype		Von Frey	Heat	Cold	Blunt pressure	Electric
*OPRM1* (μ-opioid receptor)	rs1799971 A>G	-	-	-	-	-
*OPRD1* (δ-opioid receptor)	rs1042114 T>G	0.17	*0.35*	*0.54*	***1.14***	***1.78***
	rs2234918 T>C	0.13	*0.23*	0.05	0.28	0.00
*COMT* (Cathechol-O-methyl transferase)	rs4646312 T>C	0.30	*0.26*	0.20	0.19	0.06
	rs6269 A>G	0.26	*0.35*	0.03	0.01	0.13
	rs4633 C>T	0.34	0.10	0.12	0.24	0.24
	rs4680 G>A	0.22	0.14	0.12	0.18	0.24
	rs6269G/rs4633C/4818G/rs4680G	0.26	*0.35*	0.03	0.01	0.13
	rs6269A/rs4633T/4818C/rs4680A	0.18	*0.24*	*0.25*	0.19	0.32
	rs6269A/rs4633C/4818C/rs4680G	-	-	-	-	-
	rs4646312T/rs165722T/rs6269A/rs4633T/rs4818C/rs4680A	0.24	*0.27*	0.17	0.10	0.28
	rs4646312C/rs165722C/rs6269G/rs4633C/rs4818G/rs4680G	0.23	*0.23*	0.06	0.09	0.15
	rs4646312T/rs165722C/rs6269A/rs4633C/rs4818C/rs4680G	-	-	-	-	-
*TRPV1* (Transient receptor potential cation channel, subfamily V, member 1)	rs8065080 A>G	0.16	0.13	0.03	0.06	0.10
*TRPA1* (Transient receptor potential cation channel, subfamily A, member 1)	rs11988795 G>A	0.36	0.09	0.18	0.03	0.18
	rs13255063A/rs11988795G	0.10	0.07	*0.36*	0.15	0.03
	rs13255063A/rs11988795A	0.36	0.09	0.18	0.03	0.18
	rs13255063T/rs11988795G	*0.74*	*0.37*	*0.76*	0.19	***0.97***
*FAAH* (Fatty acid amide hydrolase)	rs932816 G>A	0.06	0.09	0.09	0.40	0.27
	rs4141964 T>C	0.10	0.08	0.05	0.17	0.08
	rs2295633 G>A	0.17	0.14	0.04	0.08	0.03
	rs932816G/rs4141964T	0.37	*0.36*	*0.28*	0.08	0.16
	rs932816G/rs4141964C	0.05	0.14	0.04	0.02	0.08
	rs932816A/rs4141964T	0.06	0.09	0.09	0.40	0.27
	rs324419C/rs2295633G	0.00	0.07	0.02	0.23	0.16
	rs324419C/rs2295633A	0.06	*0.28*	*0.41*	0.35	0.46
	rs324419T/rs2295633A	*0.58*	0.19	*0.51*	0.44	0.51
*GCH1* (GTP cyclohydrolase 1)	1 particular haplotype of 3 SNPs associated to one of 15 SNPs	*0.6*	*0.72*	*0.54*	0.38	0.45
*MC1R* (Melanocortin-1 receptor)	2 variant alleles of 29insA, 451C>T, 478C>T, 479G>A, 880G>C (“red head fair skin” phenotype, n = 2)	0.06	**0.84**	**0.54**	0.05	0.03

The recessive hereditary model was used, i.e., assigning heterozygous subjects to the group of homozygous mutated carriers. The effect sizes are given in italic letters when they were larger than those of gender, and in bold letters when exceeding a value of 0.8 indicating a large effect.

**Table 4:** Effect sizes, expressed as absolute values of Cohen's d [15], of the respective factor on pain thresholds.

Factor	Polymorphism (dbSNP database number)	Effect sizes on pain thresholds (Cohen's d), dominant genetic model				
Genotype		Von Frey	Heat	Cold	Blunt pressure	Electric
*OPRM1* (μ-opioid receptor)	rs1799971 A>G	0.12	*0.20*	0.14	0.12	0.10
*OPRD1* (δ-opioid receptor)	rs1042114 T>G	0.34	0.02	*0.23*	0.07	0.16
	rs2234918 T>C	0.19	0.19	0.19	0.08	0.06
*COMT* (Cathechol-O-methyl transferase)	rs4646312 T>C	0.13	0.05	0.12	0.06	0.09
	rs6269 A>G	0.01	0.19	*0.39*	0.25	0.26
	rs4633 C>T	0.24	*0.27*	0.07	0.09	0.02
	rs4680 G>A	0.34	0.17	0.04	0.04	0.08
	rs6269G/rs4633C/4818G/rs4680G	0.07	0.08	*0.25*	0.19	0.15
	rs6269A/rs4633T/4818C/rs4680A	0.28	*0.20*	0.08	0.03	0.02
	rs6269A/rs4633C/4818C/rs4680G	*0.54*	0.01	*0.33*	0.00	0.19
	rs4646312T/rs165722T/rs6269A/rs4633T/rs4818C/rs4680A	0.29	0.19	0.02	0.03	0.04
	rs4646312C/rs165722C/rs6269G/rs4633C/rs4818G/rs4680G	0.04	0.00	0.21	0.15	0.14
	rs4646312T/rs165722C/rs6269A/rs4633C/rs4818C/rs4680G	*0.60*	0.10	0.32	0.05	0.21
*TRPV1* (Transient receptor potential cation channel, subfamily V, member 1)	rs8065080 A>G	0.04	0.19	0.11	0.16	0.24
*TRPA1* (Transient receptor potential cation channel, subfamily A, member 1)	rs11988795 G>A	0.02	0.07	0.10	0.10	0.21
	rs13255063A/rs11988795G	0.07	0.10	0.10	0.12	0.20
	rs13255063A/rs11988795A	0.02	0.07	0.10	0.10	0.21
	rs13255063T/rs11988795G	0.18	0.07	0.09	0.02	0.06
*FAAH* (Fatty acid amide hydrolase)	rs932816 G>A	0.34	0.11	0.22	0.06	0.08
	rs4141964 T>C	0.03	0.11	0.00	0.22	0.17
	rs2295633 G>A	0.0	0.07	0.02	0.23	0.16
	rs932816G/rs4141964T	0.15	0.17	0.14	0.11	0.02
	rs932816G/rs4141964C	0.03	0.11	0.00	0.22	0.17
	rs932816A/rs4141964T	0.39	0.14	0.21	0.05	0.08
	rs324419C/rs2295633G	0.17	0.14	0.04	0.08	0.03
	rs324419C/rs2295633A	0.04	0.08	*0.28*	0.12	0.09
	rs324419T/rs2295633A	0.02	0.14	0.26	0.21	0.2
*GCH1* (GTP cyclohydrolase 1)	1 particular haplotype of 3 SNPs associated to one of 15 SNPs	0.13	0.12	0.13	0.45	0.00
*MC1R* (Melanocortin-1 receptor)	2 variant alleles of 29insA, 451C>T, 478C>T, 479G>A, 880G>C (“red head fair skin” phenotype, n = 2)	-	-	-	-	-
**Gender**		0.50	0.20	0.23	**0.84**	0.68
**Sensitization**		*0.53*	***2.60***	*0.44*		

In the case of the genetic factors, the dominant hereditary model was used, i.e., assigning heterozygous subjects to the group of wild-type subjects. The effect sizes are given in italic letters when they were larger than those of gender, and in bold letters when exceeding a value of 0.8 indicating a large effect.

**Gender** produced explained 1–14.75% of the variance in the different pain thresholds (Cohen's d 0.2–0.84). The comparatively greatest fractions of the variance explained by gender were seen for blunt pressure and electric stimuli. The gender effect was directed toward higher pain sensitivity in women than in men.

**Sensitization** by capsaicin increased the pain thresholds to heat and punctate pressure (Wilcoxon tests: p<0.001) whereas menthol decreased the cold pain thresholds (Wilcoxon test: p<0.001). Heat sensitization by capsaicin explained 63% of the total variance and produced the larges effects observed in this data according to Cohen's d = 2.6. Sensitizations by capsaicin or menthol of punctate or cold pain explained 7.5 or 4.6% of the variance in those thresholds and produced medium to small effect sizes (Cohen's d = 0.53 and 0.4, respectively).

## Discussion

Pain thresholds were subject to various influences, which was most readily visible for capsaicin sensitization of heat pain perception and to a smaller extend of menthol sensitization for cold pain thresholds (Figure 1 last two lines). The basis of this large effect on heat pain thresholds is the synergistic effects of the excitations of TPRV1 by both, heat (>43°C) and capsaicin [16], [17]. As TRPV1 receptor potential channels are also considered general nocisensors [18], the effect on punctate mechanical pain has a similar explanation although the sensitization had smaller effects than on heat pain. Analogously, the effect of menthol sensitization on cold pain thresholds can be explained by a concomitant excitation of TRPM8 by both, cold stimuli between 8 and 28°C and menthol [19], [20], [21].

A part of the total variance in pain thresholds was accounted for by the subject's gender, exceeding 1/10 for blunt pressure and electrical pain stimuli. Gender effects on pain have been established for long and their present direction toward higher pain sensitivity in women than in men agrees with most studies (for reviews, see [2], [7], [8]). Explanations use sex hormones [22], [23] or differences in the function of the endogenous opioid system [24] such as a sexual dimorphism regarding opioid receptor function in rat brain structures mediating opioid analgesia [25]. Interaction of sex and genetics may follow from sex differences in the functioning of, e.g., μ- and δ-opioid receptors, COMT or FAAH [26]. Sex-differences in the response to exogenous opioids in rats were reported to depend on the genotype [27]. A sex by genotype interaction emerged for heat pain ratings with respect to the human *OPRM1* 118A>G polymorphism [28] and thermal pain sensitivity was also modulated by gender, ethnicity and psychological factors [29].

Genetic factors contributed to the explanation of the overall variance in pain thresholds. The effect sizes agreed with few elsewhere reported effect sizes such as those of variants in *COMT* or *FAAH* explaining 5–8% of the variance in experimental pain measures [30], or that of *OPRM1* rs1799971 of approximately Cohen's d = 0.3 for heat and ischemic pain thresholds or tolerance [28]. The largest genetic effect size in the present data was seen for homozygous presence of the *TRPA1* rs13255063T/rs11988795G haplotype explaining >5% of the variance in electrical pain thresholds. This genetic effect exceeded that of menthol sensitization on cold stimuli. The cold-sensitive TRPA1 receptor potential channel is mainly activated by noxious cold, chemical and endogenous irritants [31]. A decrease in cold pain withdrawal time associated with *TRPA1* rs1198795 had been observed in another study [30]. A difficulty to explain the contrasting result arises from lack of shown molecular consequences of the genetic polymorphisms. As TRPA1 is a pain sensor [18], the results with cold pain [30] point at an increased function associated with the rs1198795 variant. Increased function is conveyed by another TRPA1 mutation (N855S [32]) and associated with a rare autosomal-dominant familial syndrome characterized by episodes of debilitating upper body pain. However, the present observations of decreased pain sensitivity in carriers of a *TRPA1* haplotype, of which rs1198795 is a part, point at a decreased function of TRPA1 nocisensors associated with the frequent variants analyzed here. A few additional genetic variants modulating pain have not reached the present set of genotypes. This relates to potassium ion channels Kir3.2, for which the genetic variant *KCNJ6* rs2070995 increased opioid requirements [33], [34], and Kv_9.1_ for which the genetic *KCNS1* variants rs734784 and rs13043825 were associated with greater pain [35]. There is no strong indication that their inclusion would have changed the picture of mostly small genetic effect sizes, and information so far only shows a modulation of clinical pain including neuropathic pain but not experimental pain. The latter applies also to interleukin related genetic modulations of pain [36] and other genetically polymorphic nociceptive factors [37].

Since pain is defined as “an unpleasant sensory and emotional experience …” (International Association for the Study of Pain, http://www.iasp-pain.org), it cannot be measured directly. Correctly, the pain threshold is defined at a perceptional level as the least experience of pain which a subject can recognize. In contrast, the present pain threshold measures comprise the physical intensities of the stimuli at which the subjects indicated that they became painful. Therefore, the genetic factors, sex or sensitization have in fact modulated the lowest stimulus' strength at which the subject indicated pain. The measurement of pain has been addressed since more than half a century [38]. Quantitative information about a subject's pain may be obtained with several other methods [39]. For example, subjects may indicate the stimulus strength evoking unbearable pain (pain tolerance) or rate their pain on nominal or analog scales. In search for an objective quantification of pain, several surrogate measures have been established, such as pain-related evoked cortical electrical potentials, magnetic fields blood oxygen dependent signal, or muscle reflexes [40].

The observed small effect sizes suggest that none of the tested common factors suffices as a basis for clinical decisions or prognostic judgments with respect to pain. This may be similar for experimentally induced and clinical pain as the genetic effect of some variants has been demonstrated in both. For example, the so-called “pain-protective” *GCH1* haplotype decreased pain in healthy volunteers following administration of mechanical, heat and ischemic pain [41] or the same pain models as presently used [42], and it was associated with lower clinical pain following surgical discectomy [41] and delayed development of pain from the cancer diagnosis [43]. The poor effect size of common genetic factors is reminiscent of other multigenetic traits such as body height or type 2 diabetes, for which genome wide association studies have mainly shown that the effects of single common genetic variants on the phenotype are small [44]. This might a major reason why genetics-based pain management advices have not emerged in clinical practice [45], [46], similar to gender differences that have also raised expectations for individualized therapies and have also not entered the clinical guidelines. Therefore, an individualized pain therapy based on genotyping information is not yet imminent.

This study did not intend to reproduce genetic associations but to provide a basis for comparison of genetics' effects on pain with other effects across different studies and pain measurements. Therefore, the report was limited to effect sizes, which have become a standard part of reporting [47]. Standardized effects sizes enhanced comparison across the pain stimuli and were therefore preferred. However, if the units of measurement are meaningful on a practical level, then reporting an unstandardized measure has been advised [48]. In the present data, the difference in the physical strength of each stimulus at which it evokes pain has a practical meaning, i.e., N/m^2^ for blunt pressure or mA of 5-Hz electrical sine waves. However, the comparison across stimuli is probably more meaningful when using standardized effect sizes. This preserves the relative order of factors. However, standardizing loses the direction of the effect. The “canned” effect sizes “small”, “medium”, or “large” should not replace a decision about how large a difference is that is based on understanding of the experimental system [49].

On several pain stimuli, heat sensitization by capsaicin, gender and genetic variants produced effects on pain in the mentioned order of effect sizes (Figure 1 bottom). Reporting effect sizes is considered good practice when presenting empirical research findings in many fields and the present report may provide a basis for comparative discussions of factors influencing pain.
